# Associations of alcohol consumption with breast tissue composition

**DOI:** 10.1186/s13058-023-01638-z

**Published:** 2023-03-30

**Authors:** Lusine Yaghjyan, Yujing J. Heng, Gabrielle M. Baker, Bernard A. Rosner, Rulla M. Tamimi

**Affiliations:** 1 Department of Epidemiology, College of Public Health and Health Professions and College of Medicine, University of Florida, 2004 Mowry Rd., Gainesville, FL 32610 USA; 2 Department of Pathology, Harvard Medical School, Beth Israel Deaconess Medical Center, Boston, MA USA; 3 Channing Division of Network Medicine, Department of Medicine, Brigham and Women’s Hospital and Harvard Medical School, Boston, MA USA; 4 Department of Population Health Sciences, Weill Cornell Medicine, New York, NY USA

**Keywords:** Breast cancer, Alcohol, Benign breast disease

## Abstract

**Background:**

We investigated the associations of alcohol with percentage of epithelium, stroma, fibroglandular tissue (epithelium + stroma), and fat in benign breast biopsy samples.

**Methods:**

We included 857 cancer-free women with biopsy-confirmed benign breast disease within the Nurses’ Health Study (NHS) and NHSII cohorts. Percentage of each tissue was measured on whole slide images using a deep-learning algorithm and then log-transformed. Alcohol consumption (recent and cumulative average) was assessed with semi-quantitative food frequency questionnaires. Regression estimates were adjusted for known breast cancer risk factors. All tests were 2-sided.

**Results:**

Alcohol was inversely associated with % of stroma and fibroglandular tissue (recent ≥ 22 g/day vs. none: stroma: β = − 0.08, 95% Confidence Interval [CI] − 0.13; − 0.03; fibroglandular: β = − 0.08, 95% CI − 0.13; − 0.04; cumulative ≥ 22 g/day vs. none: stroma: β = − 0.08, 95% CI − 0.13; − 0.02; fibroglandular: β = − 0.09, 95% CI − 0.14; − 0.04) and positively associated with fat % (recent ≥ 22 g/day vs. none: β = 0.30, 95% CI 0.03; 0.57; cumulative ≥ 22 g/day vs. none: β = 0.32, 95% CI 0.04; 0.61). In stratified analysis, alcohol consumption was not associated with tissue measures in premenopausal women. In postmenopausal women, cumulative alcohol use was inversely associated with % of stroma and fibroglandular tissue and positively associated with fat % (≥ 22 g/day vs. none: stroma: β = − 0.16, 95% CI − 0.28; − 0.07; fibroglandular: β = − 0.18, 95% CI − 0.28; − 0.07; fat: β = 0.61, 95% CI 0.01; 1.22), with similar results for recent alcohol use.

**Conclusion:**

Our findings suggest that alcohol consumption is associated with smaller % of stroma and fibroglandular tissue and a greater % of fat in postmenopausal women. Future studies are warranted to confirm our findings and to elucidate the underlying biological mechanisms.

**Supplementary Information:**

The online version contains supplementary material available at 10.1186/s13058-023-01638-z.

## Introduction

Previous studies have consistently linked alcohol consumption with an increased breast cancer risk, with approximately 10–40% risk increase with 15–30 g (1–2 drinks) per day consumption and about 7% risk increase for each additional drink of alcohol [1–4]. Multiple pathways have been suggested as possible explanations for these associations, including alteration of estrogen levels, gene expression changes and carcinogenic properties of ethanol metabolites that result from their ability to form protein and DNA adducts, disrupt normal anti-oxidative defense system and DNA repair, and cause genomic instability via indirect effect on DNA methylation [3, 5].

As summarized in a recent review, in some previous studies alcohol has been associated with increased mammographic breast density, a well-established and strong breast cancer risk factor reflective of relative proportions of epithelium, stroma, and fat on the woman’s mammogram [6]. The positive associations with percent mammographic density (the proportion of epithelium and stroma [i.e., fibroglandular tissue] out of the entire breast area) were apparent in both pre- and postmenopausal women [6]. Other studies found no associations with percent density or absolute dense and non-dense areas (reflective of absolute area occupied by fibroglandular tissue and by fat, respectively) [7, 8]. The exact mechanism for the effects of alcohol on the breast tissue composition remains unclear [9]. Some of the hypothesized biological pathways that may explain the association between alcohol consumption and breast density include an increase in endogenous estrogen [10], increased aromatase activity [11], and alterations in the growth hormone insulin-like growth factor (IGF) axis [12], all of which may increase epithelial proliferation in the breast and subsequently increase breast density [13]. To the best of our knowledge, no study has examined histologic measures of breast tissue composition in normal breast tissue of cancer-free women. While radiological findings from mammograms provide information on overall relative abundance of fibrogladular structures and fat in the breast, they do not allow segmentation of epithelium from stroma. As these two tissue types have specific contributions to breast carcinogenesis, it is important to be able to consider them separately in etiological studies. In this study, we aimed to assess the associations of alcohol consumption with the extent of epithelial, stromal, fibroglandular (i.e., combined epithelium and stroma), and fat tissue in non-malignant breast tissue from benign breast biopsy samples using prospective data in cancer-free women from the Nurses’ Health Study (NHS) and Nurses’ Health Study II (NHSII) and a deep-learning computational pathology method for tissue composition assessment. The non-malignant tissue from breast biopsy samples served as a proxy for normal breast tissue. Based on the findings on positive associations of alcohol consumption and breast cancer, we hypothesized that alcohol use will be positively associated with the extent of epithelium and/or stroma.

## Materials and methods

### Study population

Our analysis included cancer-free women (controls) from the nested case–control study of breast cancer conducted among the subcohort of women with biopsy-confirmed benign breast disease (BBD) in the NHS and NHSII cohorts [14, 15]. These prospective cohorts followed registered nurses in the United States who were 30–55 years (NHS) or 25–42 years old (NHSII) at enrollment. After administration of the baseline questionnaire, the information on breast cancer risk factors (Body Mass Index [BMI], reproductive history, and postmenopausal hormones) and any diagnoses of cancer or other diseases was updated through biennial questionnaires. Cancer diagnoses were then confirmed via medical record review [16, 17]. Details of this nested case–control study and BBD assessment have been previously described [14, 15].

Early NHS questionnaires (1976, 1978, and 1980) asked whether the participant had ever been diagnosed with ‘fibrocystic disease’ or ‘other BBD’ and whether she had been hospitalized in relation to this diagnosis. Beginning in 1982, the NHS questionnaires specifically asked about a history of biopsy-confirmed BBD. The initial 1989 NHS II questionnaire and all subsequent biennial questionnaires also asked participants to report any BBD diagnosis and to indicate whether it was confirmed by biopsy or aspiration.

**Fig. 1:**
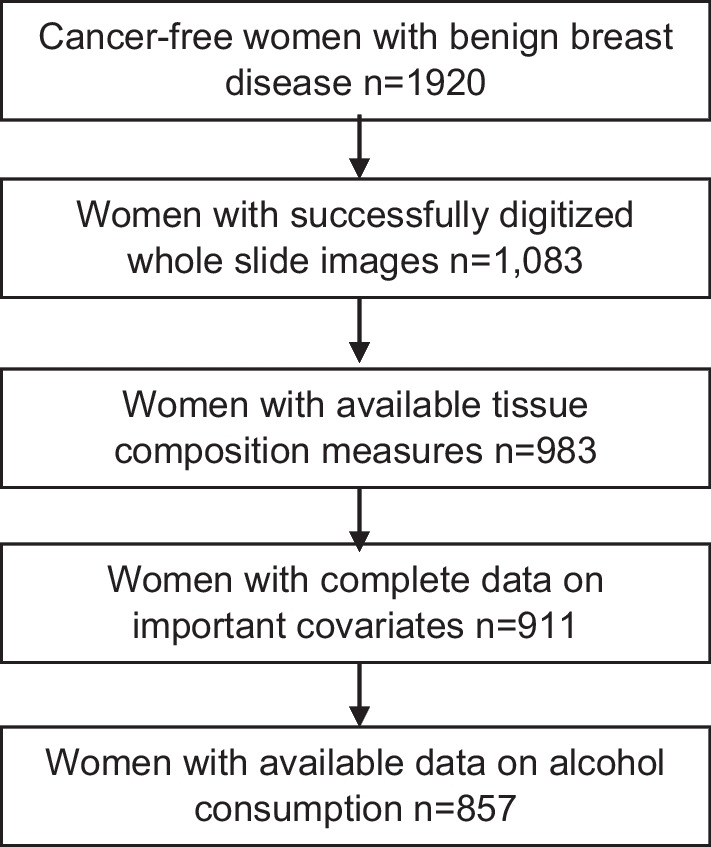
Selection of study sample

Cases were women with biopsy-confirmed BBD who reported a breast cancer diagnosis during 1976–1998 for the NHS and 1989–1999 for the NHSII following their BBD diagnosis. Using incidence density sampling, four women with biopsy-confirmed BBD who were free of breast cancer at the time of the matching case’s diagnosis (controls) were matched to the respective breast cancer case on year of birth and year of benign breast biopsy [18]. Only controls from this nested case–control study were used to examine the associations of alcohol with the tissue composition. BBD pathology records and archived biopsy specimens were obtained from the women’s hospital pathology departments. Women were excluded if they had evidence of in situ or invasive carcinoma or unknown lesion type at the time of benign breast biopsy (n = 12). Out of 1920 controls, 857 had tissue readings and information on alcohol consumption and were included in this analysis (Fig. 1). Women with and without available tissue readings had similar distributions of breast cancer risk factors.

All study procedures were in accordance with the ethical standards of the 1964 Helsinki declaration and its later amendments. The study protocol was approved by the institutional review boards of the Brigham and Women’s Hospital and Harvard T.H. Chan School of Public Health, and those of participating registries as required. Consent was obtained or implied by return of questionnaires.

### Benign breast biopsy confirmation and BBD subtypes

Hematoxylin and eosin (H&E) breast tissue slides were retrieved for biopsy-confirmed BBD patients who gave permission to review their biopsy records. The slides were independently reviewed by one of three pathologists in a blinded fashion, i.e. the evaluating pathologists were blinded to type of BBD noted on the original diagnosis [19, 20]. Any slide identified as having either questionable atypia or atypia was jointly reviewed by two pathologists. For each set of slides, a detailed work sheet was completed and the benign breast biopsy was classified according to the categories of Page et al. [21] as non-proliferative, proliferative without atypia, or atypical hyperplasia [14].

### Whole slide image acquisition

H&E slides were digitized into whole slide images at 20× or 40× using the Panoramic SCAN 150 (3DHISTECH Ltd, Budapest, Hungary). For women with good-quality slides, up to six slides from different tissue blocks were digitized. H&E slides that were not digitized were due to poor quality, slides too thick to fit into scanner, and plastic mounting coverslips. Attempts to create new H&E slides were not always possible due to missing (or returned to hospital) blocks, old-style blocks not created using tissue cassettes, or poor-quality blocks [22]. Out of all controls in the original nested case–control study (n = 1920), 1083 (or 56%) had their slides successfully digitized into whole slide images (WSI) (Fig. 1). Women with and without available tissue readings had similar distributions of breast cancer risk factors [23].

### Quantification of epithelium, stroma, and fat

Whole slide images were processed using a deep-learning computational pathology method to segment BBD tissues into epithelial, stroma, and fat regions. Tissue image analysis included normal terminal duct lobular units (TDLUs) and BBD lesions. Details of the image analysis method and its performance are described elsewhere [23, 24]. Briefly, to evaluate the tissue segmentation network, precision, recall, and Dice similarity coefficient were calculated using the held-out test set (n = 48). Dice similarity coefficient is the harmonic mean of precision (i.e., sensitivity) and recall (i.e., positive predictive value) and assesses how accurate the automated segmentation compares with ground truth on a pixel-wise basis. The range for Dice similarity coefficient is from 0 to 1, with 1 indicating perfect overlap. The majority of the precision, recall, and Dice similarity coefficient values of the tissue segmentation network and nuclei detection were > 0.75 [24]. For more details about the nuclear segmentation network, please refer to the previously published methods paper by Vellal et al. [24].

For each whole slide image, our method computed total, epithelial, stromal, and adipose tissue areas in pixels. We next calculated the average percent of each tissue type out of the total area across all available slides for each woman (median = 3, range 1–4), weighted by the total tissue area of the slides. In our sample, we observed low heterogeneity between tissue measures across available slides for a woman, with coefficients of intra-class variation ranging between 0.51 and 0.71. The approach of using weighted average to summarize whole tissue slide or core-level data into woman-level expression has been widely used in previous studies, including our own, to account for tissue composition and heterogeneity within the tissue sections/cores, to reduce the measurement error, and to reliably link tissue markers to breast cancer and its risk factors; it demonstrated high reproducibility for associations with breast cancer risk and in prognostic algorithms in clinical trials [25–33]. The distribution of breast cancer risk factors and tissue measures were similar in women with 1 versus 4 slides.

We examined associations of alcohol with percentage of each of these individual tissue regions as well as combined epithelium and stroma (fibroglandular area).

### Assessment of alcohol consumption

Information on alcohol consumption was obtained from semi-quantitative food frequency questionnaires (FFQ) [34]. In NHS, questions regarding alcohol consumption were asked in 1980, 1984, 1986, and 1990. Women reported their average consumption of beer, wine, and liquor separately in the prior year. One drink was considered equal to one can or bottle of beer, a 4-ounce glass of wine, or one drink or shot of liquor. Participants were asked to select from the following categories: almost never, 1–3 per month, 1 per week, 2–4 per week, 5–6 per week, 1 per day, 2–3 per day, 4–6 per day, ≥ 6 per day. Similarly, women in NHS II answered questions on alcohol consumption in the 1989 and 1991 questionnaires. In 1991, the questions were expanded to include red wine, white wine, light beer, regular beer, and liquor. Total alcohol consumption per questionnaire cycle was calculated as the sum of the daily number of drinks multiplied by the average alcohol content per type of alcoholic beverage (12.8 g for regular beer, 11.3 for light beer, 11.0 g for wine, and 14.0 g for liquor) [20, 35]. Alcohol consumption in these cohorts has been shown to be valid and highly reproducible in repeated assessments [36].

Women were assigned the alcohol exposure from the cycle closest to the date of the benign biopsy. If alcohol consumption was missing from the questionnaire before the biopsy date, the exposure from the preceding cycle was used (9% of women in our sample) [20]. The Spearman correlation between reported alcohol consumption in questionnaire cycles was 0.80 (*P* < 0.0001) or greater for all consecutive cycles [37]. In the current analysis, we used both a continuous (per 11 g/day [1 drink/day]) as well as categorical measure of alcohol consumption (0 [reference], < 11 g/day [< 1 drink/day], 11–< 22 g/day [1–< 2 drinks/day], and ≥ 22 g/day [≥ 2 drinks/day]). Median levels within respective categories were used for the test of trend. We also examined cumulative average alcohol consumption using all available data from before the biopsy date, with the same variable modeling approaches.

### Covariate information

Information on breast cancer risk factors was obtained from the biennial questionnaires closest to the date of the biopsy. Women were considered to be postmenopausal if they reported: (1) no menstrual periods within the 12 months before biopsy with natural menopause, (2) bilateral oophorectomy, or (3) hysterectomy with one or both ovaries retained, and were 54 years or older for ever smokers or 56 years or older for never smokers [38, 39].

### Statistical analysis

We used multivariable linear regression to examine the associations of alcohol consumption with proportion of epithelial, stromal, fibroglandular, and fat tissues. Because tissue type measures were non-normally distributed, Both visual inspection of Q–Q plots for all tissue measures and formal statistical tests demonstrated that residuals were not normally distributed (p values for all tests < 0.01); therefore, we used log-transformed values for all tissue measures in all the regression analyses to improve normality of the error distribution. The risk estimates were adjusted for age (continuous), BMI (continuous), a family history of breast cancer (yes vs. no), combined parity/age at first birth (parous with first birth before age 25, parous with first birth at or after age 25, nulliparous, unknown), age at menarche (< 12, 12, 13, > 13), menopausal status/postmenopausal hormone use (pre-, post-/no hormones, post-/past hormone use, post-/current hormone use, post-/unknown hormone use status), and study cohort (NHS, NHSII). Finally, to account for potential influence of BBD lesions on the study findings, we additionally adjusted all models for type of the BBD.

Differences in the associations of alcohol with tissue measures in pre- versus postmenopausal women were evaluated with two-way interactions and using Wald Chi-square test. We used respective medians within each of the alcohol consumption categories to model the interaction as well as the continuous alcohol consumption. All tests were 2-sided and statistical significance in all the analyses was assessed at 0.05 level. The analyses were performed using SAS software (version 9.4, SAS Institute, Cary, NC, USA).

## Results

In this study of 857 women, 260 (30.3%) had non-proliferative disease, 484 (56.5%) had proliferative disease without atypia, and 113 (13.2%) had atypical hyperplasia, consistent with previously reported distributions of these BBD subtypes [20]. The average age at the biopsy was 47 years (range 19–73 years). A majority of the women were premenopausal at the biopsy (62.5%). Age-adjusted characteristics of pre- and postmenopausal women in the study by their cumulative average alcohol consumption status are presented in Table 1. In our study, 18.1% consumed ≥ 11 g (≥ 1 drinks) of alcohol per day at the time of biopsy and 17.7% had the cumulative average consumption of ≥ 11 g (≥ 1 drinks). Recent and cumulative average alcohol use were highly correlated (correlation coefficient r = 0.90, p < 0.001). The average percentage of epithelium, stroma, and fat was 9.0% (range 0.7–52.2%), 72.4% (23.6–99.0%), and 18.6% (0–71.3%), respectively.

**Table 1:** Age-adjusted characteristics of women with biopsy confirmed benign breast disease in the Nurses’ Health Studies, by cumulative alcohol use and menopausal status

Characteristic	Premenopausal				Postmenopausal			
	None n = 159	< 11 g/day = 290	11–< 22 g/day n = 56	≥ 22 g/day n = 31	None n = 70	< 11 g/day n = 140	11–< 22 g/day n = 40	≥ 22 g/day n = 13
*Mean (SD)*								
Epithelium %	10.2 (7.5)	9.6 (6.3)	11.3 (6.7)	11.1 (5.3)	8.3 (7.7)	6.4 (4.2)	8.7 (7.5)	4.6 (1.7)
Stroma %	75.3 (11.1)	75.3 (11.0)	75.3 (11.3)	73.8 (7.0)	68.4 (10.7)	67.9 (13.9)	66.9 (10.4)	59.0 (6.8)
Fat %	14.5 (10.5)	14.9 (10.9)	13.5 (12.3)	15.2 (9.0)	23.4 (10.1)	25.7 (14.6)	24.5 (8.5)	36.5 (7.9)
Fibroglandular %^a^	85.5 (10.5)	85.1(10.9)	86.5 (12.3)	84.8 (9.0)	76.7 (10.1)	74.3 (14.6)	75.5 (8.5)	63.6 (7.9)
Age at BBD biopsy (years)^b^	39.4 (7.2)	41.7 (7.1)	42.6 (7.4)	40.2 (7.3)	58.6 (6.1)	56.8 (6.9)	57.4 (6.2)	55.4 (5.3)
Age at menarche (years)	12.7 (1.4)	12.5 (1.3)	12.6 (1.3)	12.7 (1.3)	12.6 (1.3)	12.8 (1.3)	12.6 (1.3)	12.8 (7.9)
Age at menopause (years)	NA	NA	NA	NA	49.0 (4.7)	48.3 (5.3)	48.2 (4.4)	49.0 (2.9)
Body Mass Index (kg/m^2^)	24.9 (5.2)	24.0 (4.4)	23.4 (3.6)	23.1 (3.3)	25.9 (4.4)	25.1 (4.0)	24.9 (3.7)	24.3 (1.5)
*Percentages*								
Parity/age at first birth								
Nulliparous	10	12	5	2	3	7	10	0
Parous, age < 25 years	42	43	36	59	47	48	50	60
Parous, age ≥ 25 years	46	44	59	39	49	45	40	40
Family history of BBD	8	8	19	33	18	19	13	34
Benign breast disease								
Non-proliferative	34	31	22	18	24	29	32	43
Proliferative without atypia	59	57	60	54	58	49	52	35
Proliferative with atypia	7	12	18	27	19	22	16	22
Never smoked	64	57	32	32	76	42	26	0
Past smoker	27	24	40	33	15	40	54	47
Current smoker	9	19	28	34	9	18	20	53
Never used MHT	NA	NA		NA	29	35	25	51
Used MHT in the past	NA	NA		NA	30	15	21	17
Currently using MHT	NA	NA		NA	33	39	34	26

The table does not include participants with unknown menopausal status

*SD* standard deviation; *BBD* benign breast disease; *MHT* menopausal hormone therapy; *NA* not applicable

^a^Fibroglandular tissue represents combined epithelium and stroma

^b^Value is not age adjusted

In multivariable analysis (Table 2), alcohol consumption was inversely associated with proportion of stroma and fibroglandular tissue; these associations were most pronounced for recent consumption of ≥ 22 g (≥ 2 drinks) per day (stroma: β = − 0.08, 95% Confidence Interval [CI] − 0.13; − 0.03; fibroglandular: β = − 0.08, 95% CI − 0.13; − 0.04) and cumulative average consumption of ≥ 22 g (≥ 2 drinks) per day (stroma β = − 0.08, 95% CI − 0.13; − 0.02; fibroglandular: β = − 0.09, 95% CI − 0.14; − 0.04). Alcohol consumption of ≥ 22 g/day was also positively associated with proportion of fat (β = 0.30, 95% CI 0.03; 0.57 for recent and β = 0.32, 95% CI 0.04; 0.61 for cumulative average). Alcohol was not associated with proportion of epithelium.

**Table 2:** Association of alcohol use with percentage of breast tissue composition (log-transformed) in benign breast biopsy samples (Adjusted for age (continuous), BMI (continuous), a family history of breast cancer (Yes/No), menopausal status/postmenopausal hormone use (premenopausal, postmenopausal/no hormones, postmenopausal/past hormones, postmenopausal/current hormones, postmenopausal/unknown hormone use status), age at menarche (< 12, 12, 13, > 13, unknown), combined parity/age at first birth (parous with first birth before age 25, parous with first birth at or after age 25, nulliparous, unknown), BBD category (non-proliferative, proliferative without atypia, and proliferative with atypia), and NHS cohort (NHSI, NHSII))

Alcohol use	N	Tissue type			
		% Epithelial	% Stroma	% Fat	% Fibroglandular^a^
Continuous alcohol at BBD per 11 g or one drink	816	− 0.02 (− 0.07; 0.03)	− 0.02 (− 0.03; − 0.01)	0.08 (− 0.03 × 10^–2^; 0.15)	− 0.02 (− 0.03; − 0.01)
*Categorical alcohol at BBD*					
Non-drinker	293	Ref	Ref	Ref	Ref
< 11 g/day (< 1 drink/day)	375	− 0.09 (− 0.18; 0.01)	− 0.02 (− 0.05; 0.29 × 10^–2^)	0.14 (− 0.01; 0.28)	− 0.03 (− 0.05; − 0.01)
11–< 22 g/day (1–< 2 drinks/day)	94	− 0.07 (− 0.21; 0.08)	− 0.02 (− 0.06; 0.02)	0.19 (− 0.03; 0.41)	− 0.03 (− 0.06; 0.01)
≥ 22 g/day (≥ 2 drinks/day)	54	− 0.14 (− 0.33; 0.04)	− 0.08 (− 0.13; − 0.03)	0.30 (0.03; 0.57)	− 0.08 (− 0.13; − 0.04)
p-trend	816	0.25	0.01	0.04	< 0.01
Continuous cumulative average alcohol, per 11 g or one drink	857	− 0.02 (− 0.07; 0.04)	− 0.02 (− 0.03; − 0.46 × 10^–2^)	0.07 (− 0.01; 0.15)	− 0.02 (− 0.03; − 0.01)
*Cumulative average alcohol*					
Non-drinker	245	Ref	Ref	Ref	Ref
< 11 g/day (< 1 drink/day)	460	− 0.09 (− 0.19; 0.01)	− 0.02 (− 0.04; 0.01)	0.07 (− 0.08; 0.21)	− 0.03 (− 0.05; − 0.42 × 10^–2^)
11–< 22 g/day (1–< 2 drinks/day)	104	− 0.05 (− 0.20; 0.09)	− 0.04 (− 0.08; − 0.08 × 10^–2^)	0.19 (− 0.03; 0.40)	− 0.04 (− 0.08; − 0.31 × 10^–2^)
≥ 22 g/day (≥ 2 drinks/day)	48	− 0.13 (− 0.32; 0.07)	− 0.08 (− 0.13; − 0.02)	0.32 (0.04; 0.61)	− 0.09 (− 0.14; − 0.04)
p-trend	857	0.46	< 0.01	0.01	< 0.001

^a^Fibroglandular tissue represents combined epithelium and stroma

In stratified analysis by menopausal status (Tables 3 and 4), alcohol consumption was inversely associated with proportion of stroma and fibroglandular tissue and positively associated with proportion of fat in postmenopausal women. The strongest associations were observed for recent alcohol consumption of ≥ 22 g/day (stroma: β = − 0.14, 95% CI − 0.24; − 0.05; fibroglandular: β = − 0.16, 95% CI − 0.25; − 0.08; fat: β = 0.79, 95% CI 0.28; 1.31) and cumulative average consumption of ≥ 22 g/day (stroma β = − 0.16, 95% CI − 0.28; − 0.05; fibroglandular: β = − 0.18, 95% CI − 0.28; − 0.07; fat: β = 0.61, 95% CI 0.01; 1.22). Alcohol consumption was not associated with tissue measures in premenopausal women. We found significant interactions of alcohol consumption with menopausal status for recent continuous alcohol consumption in relation to fibroglandular tissue (p-interaction = 0.02) and interactions of continuous cumulative alcohol use with menopausal status in relation to stroma (p-interaction = 0.02), fat (p-interaction = 0.01), and fibroglandular tissue (p-interaction = 0.01) that were driven by positive associations in postmenopausal women and no associations observed among premenopausal women. A few other interactions between menopausal status and alcohol consumption also showed marginal significance (Additional file 1: Table S1).

**Table 3:** Association of alcohol use with percentage of breast tissue composition (log-transformed) in benign breast biopsy samples, premenopausal controls only (Adjusted for age (continuous), BMI (continuous), a family history of breast cancer (Yes/No), age at menarche (< 12, 12, 13, > 13, unknown), combined parity/age at first birth (parous with first birth before age 25, parous with first birth at or after age 25, nulliparous, unknown), BBD category (non-proliferative, proliferative without atypia, and proliferative with atypia), and NHS cohort (NHSI, NHSII))

Alcohol use	N	% Epithelial	% Stroma	% Fat	% Fibroglandular^a^
Continuous alcohol at BBD per 11 g or one drink	511	0.01 (− 0.05; 0.07)	− 0.01 (− 0.03; 0.01)	0.02 (− 0.08; 0.11)	− 0.01 (− 0.02; 0.01)
*Categorical alcohol at BBD*					
Non-drinker	178	Ref	Ref	Ref	Ref
< 11 g/day (< 1 drink/day)	246	− 0.09 (− 0.20; 0.02)	0.46 × 10^–2^ (− 0.03; 0.04)	− 0.01 (− 0.18; 0.16)	− 0.27 × 10^–2^ (− 0.03; 0.02)
11–< 22 g/day (1–< 2 drinks/day)	55	− 0.06 (− 0.24; 0.12)	0.01 (− 0.04; 0.06)	0.08 (− 0.19; 0.35)	− 0.01 (− 0.05; 0.04)
≥ 22 g/day (≥ 2 drinks/day)	32	− 0.24 × 10^–2^ (− 0.22; 0.22)	− 0.05 (− 0.11; 0.02)	0.05 (− 0.29; 0.39)	− 0.03 (− 0.09; 0.02)
p-trend	511	0.83	0.20	0.57	0.26
Continuous cumulative average alcohol, per 11 g or one drink	536	0.01 (− 0.05; 0.08)	− 0.01 (− 0.02; 0.01)	− 0.49 × 10^–2^ (− 0.10; 0.09)	− 0.36 × 10^–2^ (− 0.02; 0.01)
*Cumulative average alcohol*					
Non-drinker	159	Ref	Ref	Ref	Ref
< 11 g/day (< 1 drink/day)	290	− 0.06 (− 0.17; 0.06)	− 0.01 (− 0.04; 0.02)	0.03 (− 0.14; 0.20)	− 0.01 (− 0.04; 0.02)
11–< 22 g/day (1–< 2 drinks/day)	56	− 0.05 (− 0.23; 0.14)	− 0.02 (− 0.07; 0.03)	0.10 (− 0.17; 0.37)	− 0.03 (− 0.07; 0.02)
≥ 22 g/day (≥ 2 drinks/day)	31	− 0.03 (− 0.26; 0.20)	− 0.03 (− 0.10; 0.03)	0.15 (− 0.19; 0.50)	− 0.04 (− 0.09; 0.02)
p-trend	536	0.95	0.26	0.31	0.14

^a^Fibroglandular tissue represents combined epithelium and stroma

**Table 4:** Association of alcohol use with percentage of breast tissue composition (log-transformed) in benign breast biopsy samples, postmenopausal controls only (Adjusted for age (continuous), BMI (continuous), a family history of breast cancer (Yes/No), postmenopausal hormone use (none, past, current, unknown), age at menopause (< 46, 46–< 50, 50–< 55, ≥ 55, unknown), age at menarche (< 12, 12, 13, > 13, unknown), combined parity/age at first birth (parous with first birth before age 25, parous with first birth at or after age 25, nulliparous, unknown), BBD category (non-proliferative, proliferative without atypia, and proliferative with atypia), and NHS cohort (NHSI, NHSII)

Alcohol use	N	% Epithelial	% Stroma	% Fat	% Fibroglandular^a^
Continuous alcohol at BBD per 11 g or one drink	254	− 0.05 (− 0.16; 0.06)	− 0.04 (− 0.07; − 0.01)	0.21 (0.05; 0.37)	− 0.04 (− 0.07; − 0.01)
*Categorical alcohol at BBD*					
Non-drinker	96	Ref	Ref	Ref	Ref
< 11 g/day (< 1 drink/day)	108	0.02 (− 0.17; 0.22)	− 0.10 (− 0.15; − 0.05)	0.47 (0.19; 0.76)	− 0.09 (− 0.14; − 0.04)
11–< 22 g/day (1–< 2 drinks/day)	32	0.09 (− 0.19; 0.37)	− 0.08 (− 0.15; − 0.05)	0.40 (− 0.01; 0.81)	− 0.07 (− 0.14; − 0.11 × 10^–2^)
≥ 22 g/day (≥ 2 drinks/day)	18	− 0.31 (− 0.66; 0.04)	− 0.14 (− 0.24; − 0.05)	0.79 (0.28; 1.31)	− 0.16 (− 0.25; − 0.08)
p-trend	254	0.16	0.02	0.01	< 0.01
Continuous cumulative average alcohol, per 11 g or one drink	263	− 0.02 (− 0.13; 0.09)	− 0.05 (− 0.08; − 0.02	0.22 (0.05; 0.39)	− 0.05 (− 0.08; − 0.02)
*Cumulative average alcohol*					
Non-drinker	70	Ref	Ref	Ref	Ref
< 11 g/day (< 1 drink/day)	140	− 0.05 (− 0.25; 0.14)	− 0.05 (− 0.10; 0.01)	0.13 (− 0.16; 0.43)	− 0.06 (− 0.11; − 0.01)
11–< 22 g/day (1–< 2 drinks/day)	40	0.05 (− 0.22; 0.31)	− 0.07 (− 0.15; − 0.01 × 10^–2^)	0.32 (− 0.08; 0.71)	− 0.05 (− 0.12; 0.01)
≥ 22 g/day (≥ 2 drinks/day)	13	− 0.14 (− 0.54; 0.27)	− 0.16 (− 0.28; − 0.05)	0.61 (0.01; 1.22)	− 0.18 (− 0.28; − 0.07)
p-trend	263	0.95	< 0.01	0.03	< 0.01

^a^Fibroglandular tissue represents combined epithelium and stroma

## Discussion

In this study of 857 cancer-free women, alcohol consumption was inversely associated with proportion of stroma and fibroglandular tissue and positively associated with fat, with more pronounced effects for recent consumption of ≥ 22 g/day (≥ 2 drinks/day) and cumulative average consumption of ≥ 22 g/day (≥ 2 drinks/day). In stratified analysis by menopausal status, these associations persisted in postmenopausal women only.

We found an inverse association of alcohol with stroma and fibroglandular tissue. Previous studies of alcohol and mammographic breast density generally have shown positive associations [6]. The findings in postmenopausal women have been conflicting [40, 41]. Mammographic breast density is more reflective of alterations in stromal composition rather than epithelium [42]. In contrast to our a priori hypothesis, our results do not suggest that alcohol could influence breast density by its influence on stroma. However, our results are based on a direct measurement of biopsy sample retrieved from a specific area of the breast while studies of mammographic density take into account the overall breast density pattern of the entire breast or the average density measurements of both breasts. Second, some of the previous studies are based on radiologist-assisted density estimation while our study employs computerized assessment of breast histopathological images.

In terms of the potential mechanisms behind alcohol-associated increase in breast cancer risk, by reducing the extent of stroma, alcohol may be potentially suppressing the protective role of stroma in sustaining normal breast tissue structure and function via a variety of signaling mechanisms that control and regulate normal processes and suppress malignant transformation [43–47]. So these findings could potentially offer new and previously unrecognized mechanisms of alcohol’s contributions to breast carcinogenesis such as epithelial-stromal interactions. However, future studies are warranted to confirm our findings and to elucidate biological mechanisms behind the observed associations.

We also observed positive associations of alcohol with the proportion of fat. In our earlier study of alcohol and breast density in postmenopausal women from another nested case–control study within NHS/NHSII, we reported an inverse association of alcohol with absolute non-dense area on the mammogram, which is represented by adipose tissue [48]. Similar association was also observed in another study in postmenopausal women [41]. This inverse association could potentially be explained by alcohol’s fat-reducing effect on various tissues, including breast, due to high energy demanding nature of the microsomal ethanol oxidation which dominates in women [49] as well as alcohol-associated increase in thermogenesis [41, 50]. However, our findings from the current study with direct measurement of tissue composition suggests a greater proportion of fat in women with greater alcohol consumption limited to postmenopausal women. During menopausal transition, breast tissue undergoes significant remodeling with involution of breast lobules with a reduction in the number and size of the acini per lobule and replacement of the intralobular stroma with dense collagen and, eventually, fatty tissue [51]. Some studies in animal models also suggest that estrogen may promote involution by exacerbating inflammation, cell death and adipocytes repopulation [52]. Thus, the associations observed in postmenopausal women could potentially be reflective of the effects of alcohol on involution, via either direct or estrogen-mediated mechanisms. Additionally, much higher levels of estrogens in premenopausal as compared to postmenopausal women may have dominating effects on breast tissue composition as compared to any effects of alcohol. These conflicting results could potentially be explained by the reasons noted above.

To our knowledge, this is the first study to explore the associations of alcohol with the proportion of epithelium, stroma, fibroglandular, and fat tissues using breast histopathological images. The analysis used data from the NHS and NHSII, established cohorts with more than 30 years of follow-up, confirmed benign breast disease status, and comprehensive information on breast cancer risk factors. Our study has a few limitations. Despite the prospective nature of the cohort, potential errors in reporting of alcohol consumption are possible. However, previous validation studies suggest reasonable reproducibility and validity of the data from food frequency questionnaires for the use in studies of associations between diet and health outcomes in epidemiologic studies [53, 54]. High accuracy in self-reported alcohol consumption has been reported in both men and women, including the NHS [36, 55, 56]. There was a high correlation between alcohol intake reported on FFQ and that assessed by multiple week diet records over the same period (r = 0.90). Moreover, four years after completing the diet record, another assessment was done to collect self-reported alcohol intake over the previous 4 years. These measures were highly correlated as well (r = 0.84). This evidence suggests that a FFQ provides reliable and sufficiently accurate information on alcohol intake over an extended period of time for use in epidemiologic investigations [36]. Next, breast tissue composition was measured on a whole tissue section from a biopsy which represents a smaller piece of tissue and it is unclear how generalizable it is to the rest of the breast, which may also explain the differences in our findings as compared to those for associations between alcohol and mammographic breast density. However, our previous work demonstrates that this sampling approach still provides strong evidence for a priori hypotheses and meaningful findings for breast tissue involution [57], identification of markers associated with breast cancer risk [14, 26, 58], and associations with known breast cancer risk factors, suggesting that this limitation has minimal impact on research findings [59]. Further, a study from Mayo BBD cohort has shown a large concordance in lobular involution across all four quadrants of the breast [51], suggesting low heterogeneity and thus minimum potential measurement error. Finally, our study includes only cancer-free women with clinically-indicated biopsy resulting in BBD diagnosis and since the analysis of the whole slide images included both the background normal tissue and benign lesions, the findings are expected to be generalizable to cancer-free women with BBD.

## Conclusions

Our findings suggest that alcohol consumption is associated with smaller proportion of stroma and fibroglandular tissue and a greater proportion of fat in postmenopausal women. Future studies are warranted to confirm our findings and to elucidate the underlying biological mechanisms.

## Supplementary Information

**Additional file 1. Table S1.** Interactions of alcohol with menopausal status in relation to proportion of breast tissue composition (*p*-values).

## Data Availability

Data are available upon reasonable written request. According to standard controlled access procedure, applications to use NHS/NHSII/HPFS resources will be reviewed by our External Collaborators Committee for scientific aims, evaluation of the fit of the data for the proposed methodology, and verification that the proposed use meets the guidelines of the Ethics and Governance Framework and the consent that was provided by the participants. Investigators wishing to use NHS/NHSII/HPFS data are asked to submit a brief description of the proposed project (go to https://www.nurseshealthstudy.org/researchers (contact email: nhsaccess@channing.harvard.edu) and https://sites.sph.harvard.edu/hpfs/for-collaborators/for details.

## Abbreviations

BBD: Benign breast disease
BMI: Body Mass Index
CI: Confidence interval
FFQ: Food frequency questionnaire
H&E: Hematoxylin and eosin
NHS: Nurses’ Health Study
