# Supplementary material for: Associations of alcohol consumption with breast tissue composition
Source: Breast Cancer Res. 2023 Mar 30;25:33. doi: 10.1186/s13058-023-01638-z (PMC10061845; doi:10.1186/s13058-023-01638-z)
Supplement: Supplementary file 1 — Additional file 1. Table S1. Interactions of alcohol with menopausal status in relation to proportion of breast tissue composition (p-values). [file 13058_2023_1638_MOESM1_ESM.docx]

**Additional file 1: Table S1. Interactions of alcohol with menopausal status in relation to proportion of breast tissue composition (p-values)**

| **Alcohol use** | **% Epithelial** | **% Stroma** | **% Fat** | **% Fibroglandular^a^** |
| --- | --- | --- | --- | --- |
| Continuous alcohol at BBD (drinks/day) | 0.15 | 0.15 | 0.04 | 0.02 |
| Categorical alcohol at BBD (non-drinker, <11 g/day (<1 drink/day), 11-<22g/day (1-<2 drinks/day), ≥ 22 g/day (≥2 drinks/day)) | 0.10 | 0.32 | 0.08 | 0.06 |
| Continuous cumulative average alcohol (drinks/day) | 0.31 | 0.02 | 0.01 | 0.01 |
| Cumulative average alcohol (non-drinker, <11 g/day (<1 drink/day), 11-<22g/day (1-<2 drinks/day), ≥ 22 g/day (≥2 drinks/day) | 0.68 | 0.07 | 0.12 | 0.07 |
|  |  |  |  |  |

^a^Fibroglandular tissue represents combined epithelium and stroma
